# Blood pressure control and left ventricular echocardiographic progression in hypertensive patients: an 18-month follow-up study

**DOI:** 10.3389/fcvm.2023.1161993

**Published:** 2023-07-26

**Authors:** Yan Yang, Yan Li, Limin Zhu, Jianzhong Xu, Xiaofeng Tang, Pingjin Gao

**Affiliations:** ^1^ Department of Cardiovascular Medicine, State Key Laboratory of Medical Genomics, Shanghai Key Laboratory of Hypertension, Shanghai Institute of Hypertension, Ruijin Hospital, Shanghai Jiao Tong University School of Medicine, Shanghai, China; ^2^ Laboratory of Vascular Biology, Institute of Health Sciences, Shanghai Institutes for Biological Sciences, Chinese Academy of Sciences, Shanghai, China

**Keywords:** hypertension, global longitudinal strain, left ventricular mass index, blood pressure control, echocardiography

## Abstract

**Objectives:**

The impact of blood pressure (BP) control and its timing on left ventricular (LV) structure and function remains unclear. The present study was to evaluate whether BP control correlated with conventional LV geometry and function indexes or global longitudinal strain (GLS) progression, and when echocardiographic changes would occur in essential hypertension.

**Methods and results:**

A total of 62 participants (mean age 55.2 ± 11.5, male 71.0%) with uncontrolled hypertension were enrolled in the longitudinal study. Patients were followed up at the 6-month and 18-month, when echocardiographic measurements were performed and BP control was evaluated during the follow up period. At the 6- and 18-month examination, we divided the hypertensive patients into two groups as BP controlled and uncontrolled group. Patients with BP uncontrolled (*n* = 33) had higher LV mass index (*P* = 0.02), higher left atrial volume index (*P* = 0.01), worse GLS (*P* = 0.005) and GLS changes (*P* = 0.003) compared with controlled BP (*n* = 29) at the 6-month follow-up examination. Patients with uncontrolled BP (*n* = 25) had higher LV mass index (*P* = 0.001), higher LV mass index changes (*P* = 0.01), higher relative wall thickness (*P* = 0.01), higher *E*/*e*′ (*P* = 0.046), worse GLS (*P* = 0.02) and GLS changes (*P* = 0.02) compared to BP controlled group (*n* = 24) at the 18-month follow-up examination. GLS changes were associated with BP control (*β *= 0.370, *P* = 0.004 at the 6-month examination and *β *= 0.324, *P* = 0.02 at the 18-month examination, respectively) in stepwise multivariate regression analysis. LV mass index changes was corelated with systolic BP (*β *= 0.426, *P* = 0.003) at the 18-month follow-up examination in stepwise multivariate regression analysis. Neither was GLS changes nor LV mass index changes were related to antihypertensive medication class, including combination therapy in 6- or 18-month follow up examination.

**Conclusions:**

Our findings offer new clinical evidence on the association of BP control with echocardiographic changes in hypertensive patients, and, in particular, support the view that GLS progression was earlier and subtler than conventional LV geometry and function parameters. GLS changes were significant between BP controlled and uncontrolled patients even in 6-month follow-up period.

## Introduction

Hypertension is both a most prevalent cardiovascular disease and a most significant cause of cardiovascular mortality. In China, hypertension affects about 30 percent of adults, but blood pressure control rate is only 16.8% according to the 2018 revised Chinese guidelines for the management of hypertension (1). BP control showed a robust association with increased risk of cardiovascular disease and chronic kidney disease, as well as target organ damage progression in hypertension, which was confirmed by previous studies and guidelines (1–3).

Conventional echocardiography is not able to detect early subtle abnormalities caused by the hemodynamic changes of hypertension. As a new method for assessing LV systolic function, the speckle tracking echocardiography, assessed as global longitudinal strain (GLS) can identify subtle adaptive changes in asymptomatic hypertensive patients (4, 5). Many researchers found the absolute value of GLS was significantly lower in hypertensive patients (6, 7). The improvement of conventional echocardiographic parameters for LV geometry and function (8–11) as well as LV GLS after antihypertensive treatment (8, 12–16) were widely reported. Many studies reported the improvement in GLS associated with the use of antihypertensive medications. However, the consensus about the timing of these changes were not reported, and neither was the relationship between BP reduction or the antihypertensive medication class and GLS well established. Therefore, we prospectively investigated whether BP control was correlated with GLS or the conventional echocardiographic parameters improvement and the timing of these changes occurring in hypertensive patients for 18-month follow-up period.

## Methods

### Patient population

Between September 2013 and December 2015, 80 uncontrolled hypertensive patients were recruited from the department of hypertension and outpatient clinic of Ruijin Hospital, Shanghai, China. All the participants provided prior written informed consent for participation in this study. This study was approved by the Ethics Committee of Ruijin Hospital. Exclusion criteria included recent acute coronary syndrome (within 6 months), active myocarditis, significant valvular heart disease, uncontrolled arrhythmia, acute heart failure, secondary hypertension, peripheral artery disease and white coat hypertension.

Of the 80 participants, 62 participants (mean age 55.2 ± 11.5, male 71.0%) were followed up from the initial medical visit to the 6-month examination, 12 patients declined to further participate in the study and 6 patients were diagnosed as white coat hypertension by ambulatory blood pressure monitoring (ABPM). All the 62 participants were willing to be reexamined for the 6-month follow-up echocardiographic examination and clinical data recollection. Among them, 56 subjects accepted the 18- month follow up examination, from April 2015 to December 2016, for a total time interval of 12.8 ± 1.5 months from the second medical visit, of which 49 had a valuable echocardiographic examination.

### Clinical evaluation

All participants provided their medical history and standardized questionnaires were used to determine medication use, and cardiovascular risk assessment at baseline, at the 6-month, and at the 18-month follow-up medical visit. Clinical examination, including echocardiography were performed at the three times of medical visits. The hypertensive patients were defined as systolic blood pressure >130 mmHg and/or diastolic blood pressure >80 mmHg by ABPM or use of antihypertensive agents for controlling BP at the initial medical visit. Blood pressure (BP) control was defined as blood pressure less than 140/90 mmHg at office and/or less than 135/85 mmHg at home during the follow up period.

Validated oscillometric SpaceLabs 90,217 monitors (Space-Labs Inc., Redmond, Washington, USA) were used for ABPM measurement in the follow-up study. They were programmed to obtain BP readings at 20 min intervals in daytime (from 06:00 to 22:00) and at 30 min intervals at night (from 22:00 to 06:00). Omron HEM-7051 monitors (Omron Health Care, Kyoto, Japan) were used for clinic and home BP measurements in the study. Three consecutive BP readings were obtained according to the recommendation of the Chinese guidelines for the management of hypertension after the participants had rested in the sitting position for more than 5–10 min. The three readings were averaged for analysis afterwards.

### Echocardiography

#### Transthoracic 2D data acquisitions

Echocardiographic data were collected with a commercially available instrument (E9, GE Health Care, Milwaukee, WI) according to standard procedures. LV diameters, septal and posterior wall thickness were measured according to the guidelines of the American Society of Echocardiography from 2-dimensionally guided M-mode tracings, with a recording speed at 50–100 cm/s (17). LV mass was calculated from M-mode echocardiograms according to the formula described by Devereux et al. (18). LV mass index (LVMI) in g/m^2^ was indexed from LV mass to body surface area. LA volume index (LAVI) in g/m^2^ was indexed from left atrial volume to body surface area. The peak early (E) and late (A) transmitral flow velocities, the ratio of early to late peak velocities (E/A) were measured from apical 4-chamber view, by placing a pulsed-wave (PW) Doppler sample volume between mitral leaflet tips during diastole. The deceleration time of E velocity and isovolumic relaxation time (IVRT) were measured from apical 5-chamber view by placing a continues wave Doppler sample volume between the LV out flow and inflow tract. The IVRT measurement was estimated from the cessation of the aortic flow and the onset of transmitral inflow. The relative wall thickness (RWT) was calculated as the ratio of two times posterior wall thickness to LV diastolic diameter.

Tissue Doppler imaging (TDI) was assessed by means of mitral annulus in four-chamber view. Tissue Doppler sample volume was placed at the septal and lateral sides of the mitral annulus to determine the early diastolic annular (*e*′) from the TDI recordings. The ratio of peak early (*E*) to tissue Doppler early peak diastolic (*e*′) was calculated. The average values of septal and lateral ratios were calculated for the assessment of global LV diastolic function (19).

### Analysis of global longitudinal strain (GLS)

The speckle tracking data were analyzed off line using dedicated automated software (Echo PAC ultrasound workstation, Version 203; GE Health Care, Milwaukee, WI). Three endocardial markers were placed in an end-diastolic frame at apical 4-chamber, 2-chamber and 3-chamber views for 2D longitudinal speckle tracking analysis. The longitudinal strain was obtained by the myocardial motion of each segment at each cardiac cycle in the region of interest tracked frame by frame, and the corresponding curve was measured by the software automatically as well. Adequate tracking could be verified in real time, which was corrected by adjusting the region of interest or correcting the contour to ensure optimal tracking by manually. Finally, the left ventricular GLS was calculated based on the average value derived from all segments' longitudinal strain.

To calculate the intra-observer and inter-observer left ventricular GLS variability, the speckle tracking parameters were remeasured in the first 13 patients by the same observer 4 weeks after the initial evaluation and two different observers without reviewing the patients' previous reports. The intra-observer and inter-observer variation coefficients of left ventricular GLS were 1.5% and 3.8%, respectively.

The respective differences (Δ) in all the measured echocardiographic parameters, including left ventricular GLS between the baseline and at the 6-month, as well as 18- month follow-up examination was calculated.

### Statistical analysis

We use G*Power 3.1.9.7 to estimate the sample size, given *α*, power and effect size, the total sample size is 56. Data were stored and analyzed using the SPSS 23.0 statistical package (SPSS Inc, Chicago, IL). Continuous data were presented as mean ± SD and the frequencies of subjects in each category were presented as categorical variables. Comparison between different studied groups were performed by independent *t*-tests or paired *t*-tests. Comparisons of categorical variables among groups were performed using *χ*^2^ test. Associations among continuous and categorical variables were assessed using linear regression analysis and Pearson correlation analysis. Association with the GLS changes and LVMI changes were determined by multivariate linear regression analysis with forward selection followed by backward elimination of covariates, which only resulted with significantly increased predictability of the dependent variable. Age, sex, body mass index (BMI), BP control, diabetes mellitus, hyperlipoidemia, systolic and diastolic blood pressure were selected as independent variables, the GLS changes during 6-month or 18-month follow-up period was the dependent variable. Age, sex, BMI, BP control, diabetes mellitus, hyperlipoidemia, systolic and diastolic blood pressure, usage of ACE-I or ARB were selected as independent variables, the LVMI changes during 18-month follow-up period was the dependent variable. The GLS changes and LVMI changes were entered as continuous values in the model. A 2- tailed *P* value of <0.05 was defined as statistically significant, and a *P* value of <0.01 as highly significant.

## Results

### Patient characteristics

The present analysis included 62 essential hypertensive patients (mean age 55.2 ± 11.5 years, 71.0% men) with complete data and measurable echocardiographic variables of interest at baseline and at the 6-month follow-up analysis. The patients were classified into two groups based on their BP control during the 6-month follow up period as controlled group (*n* = 29, 46.8%) and uncontrolled group (*n* = 33, 53.2%). Among the 49 patients who had echocardiographic examination at the third medical visit, 21 patients had consistently uncontrolled BP at the 18-month and 6-month visits, 18 patients had consistently controlled BP at the 18-month and 6-month visits, 6 patients had controlled BP at the 18-month visit and uncontrolled BP at the 6-month visit and 4 patients had uncontrolled BP at the 18-month visit but had controlled BP at the 6-month visit. Among the 13 patients who did not receive echocardiographic measurement at the 18-month visit, 6 patients were uncontrolled and 7 were controlled patients at the 6-month visit.

Baseline clinical data of the hypertensive patients were summarized in Table 1. Of the 62 patients, there was no difference in age, sex, current smoker, current alcohol intake, BMI, systolic or diastolic blood pressure, hypertension course, diabetes mellitus, hyperlipoidemia, chronic kidney disease, proteinuria, stroke, the usage of antihypertensive medications, as well as combination therapy or statin between BP controlled and uncontrolled groups at baseline. No coronary artery disease history was reported by the patients in this study (Table 1).

**Table 1:** Patients' clinical characteristics at baseline.

	Controlled group	Uncontrolled group	*P* value
	(*n* = 29)	(*n* = 33)	
Age (year)	53.3 ± 12.3	57.6 ± 9.9	0.14
Sex, male, *n* (%)	21 (72.4)	23 (69.7)	0.81
Current smoker, *n* (%)	10 (34.5)	15 (45.5)	0.50
Current alcohol intake, *n* (%)	4 (13.8)	11 (33.3)	0.07
Body mass index (kg/m^2^)	27.2 ± 3.8	26.2 ± 3.3	0.24
Systolic blood pressure (mmHg)	155.1 ± 8.2	158.5 ± 11.2	0.19
Diastolic blood pressure (mmHg)	90.7 ± 9.1	91.9 ± 11.6	0.66
Hypertension course (years)	13.5 ± 11.6	16.9 ± 11.2	0.27
Diabetes mellitus, *n* (%)	6 (20.7)	10 (30.3)	0.39
Hyperlipoidemia, *n* (%)	8 (27.6)	11 (33.3)	0.62
Proteinuria, *n* (%)	6 (20.7)	4 (12.1)	0.36
Chronic kidney disease, *n* (%)	3 (10.3)	2 (6.1)	0.54
Stroke, *n* (%)	4 (13.8)	5 (15.2)	0.88
Anti-hypertensive drugs			
ACE-I, *n* (%)	3 (10.3)	7 (21.2)	0.25
ARB, *n* (%)	17 (58.6)	21 (63.6)	0.69
CCB, *n* (%)	24 (82.8)	30 (90.9)	0.34
Beta-blocker, *n* (%)	10 (34.5)	17 (51.5)	0.18
Diuretic, *n* (%)	10 (34.5)	14 (42.2)	0.52
Combination therapy (%)	22 (75.8)	30 (90.9)	0.11
Statin, *n* (%)	5 (17.2)	7 (21.2)	0.69

ACE-I, medication with angiotensin-converting enzyme (ACE) inhibitor; ARB, medication with angiotensin receptor blocker; CCB, medication with calcium channel blocker.

### Predictive value of blood pressure control for left ventricular progression

**Figure 1:**
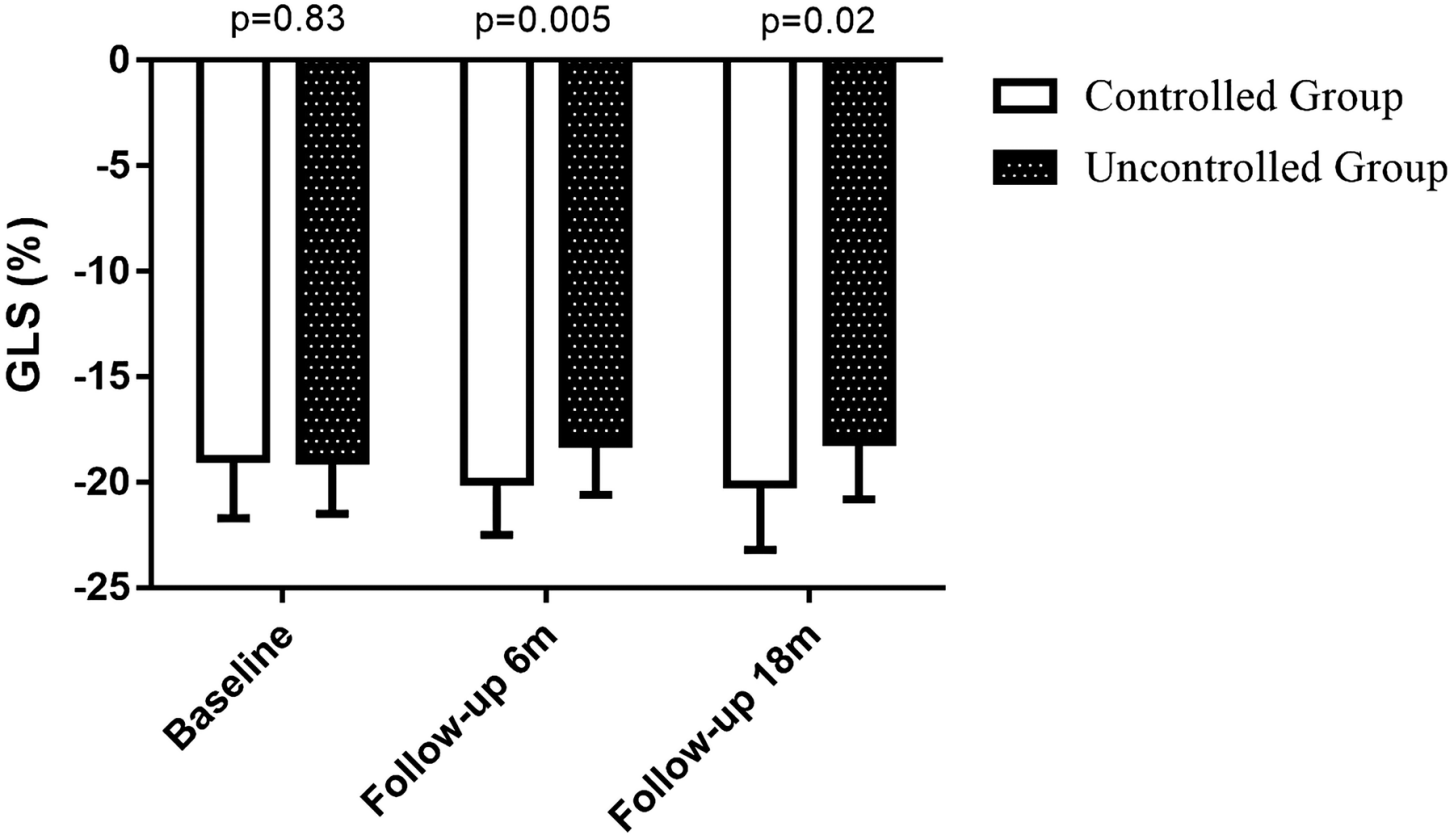
GLS at baseline, 6-month follow-up visit and 18-month follow-up visit. GLS at baseline: controlled group (*n* = 29): −18.9 ± 2.8 vs. uncontrolled group (*n* = 33): −19.0 ± 2.5, *P* = 0.83; GLS at 6-month follow-up visit: controlled group (*n* = 29): −20.0 ± 2.5 vs. uncontrolled group (*n* = 33): −18.2 ± 2.4, *P* = 0.005; GLS at 18-month follow-up visit: controlled group (*n* = 24): −20.1 ± 3.1 vs. uncontrolled group (*n* = 25): −18.8 ± 3.5, *P* = 0.02.

At baseline, patients had similar left ventricular geometric and systolic function, as well as right heart function, in uncontrolled group as in controlled group (Table 2). Moreover, there was no difference in the GLS between two groups at baseline (Figure 1). Patients in uncontrolled group had higher LAVI (28.8 ± 5.0 vs. 26.1 ± 5.0, *P* = 0.048) and longer IVRT (111 ± 27 vs. 94 ± 15, *P* = 0.003) than controlled group, but there was no difference in E/A ratio, deceleration time, *e*′ or average *E*/*e*′ between groups at baseline (Table 2).

**Table 2:** Patients' echocardiographic characteristics at baseline and 6 months follow-up visit.

	Baseline			6 months follow-up		
	Controlled group	Uncontrolled group	*P* value	Controlled group	Uncontrolled group	*P* value
	(*n* = 29)	(*n* = 33)		(*n* = 29)	(*n* = 33)	
Left ventricular geometric and systolic function parameters						
LV end-diastolic diameter (mm)	50.0 ± 4.6	50.1 ± 4.4	0.94	49.6 ± 4.3	50.2 ± 4.2	0.57
LV end-systolic diameter (mm)	30.5 ± 4.7	29.9 ± 4.3	0.64	29.7 ± 3.7	30.0 ± 3.4	0.76
LV end-diastolic volume (ml)	120.2 ± 25.7	120.2 ± 23.9	0.99	117.4 ± 23.3	120.4 ± 22.6	0.62
LV end-systolic volume (ml)	37.9 ± 14.6	36.2 ± 13.1	0.63	34.8 ± 10.8	35.9 ± 9.8	0.69
LV ejection fraction (%)	69.1 ± 6.9	70.5 ± 5.7	0.38	70.6 ± 6.0	70.3 ± 5.3	0.83
Interventricular septum (mm)	11.3 ± 1.6	11.8 ± 1.8	0.25	11.1 ± 1.5	11.8 ± 1.9	0.10
LV posterior wall (mm)	10.8 ± 1.6	10.8 ± 1.1	0.95	10.4 ± 1.3	11.0 ± 1.3	0.06
LV mass (g)	214.3 ± 65.8	218.4 ± 42.7	0.77	200.8 ± 51.4	222.5 ± 53.4	0.11
LV mass index (g/m^2^)	105.4 ± 29.1	116.7 ± 29.5	0.14	99.2 ± 25.0	118.5 ± 35.2	0.02
Relative wall thickness	0.43 ± 0.05	0.44 ± 0.06	0.84	0.42 ± 0.05	0.44 ± 0.06	0.13
Heart rate (bpm)	68.1 ± 7.3	65.7 ± 8.5	0.25	68.8 ± 10.7	68.4 ± 9.3	0.65
Left ventricular diastolic function parameters						
Left atrial volume index (ml/m^−2^)	26.1 ± 5.0	28.8 ± 5.0	0.048	24.8 ± 4.1	27.6 ± 4.7	0.01
Early: late-wave ratio	0.94 ± 0.26	0.87 ± 0.24	0.26	0.91 ± 0.24	0.91 ± 0.24	0.91
Early wave deceleration time (ms)	218 ± 38	222 ± 42	0.69	214 ± 37	225 ± 61	0.38
IVRT (ms)	94 ± 15	111 ± 27	0.003	98 ± 17	105 ± 22	0.19
*e*′ (cm/s)	6.8 ± 2.0	6.0 ± 1.6	0.06	7.2 ± 2.2	6.2 ± 1.6	0.04
*E*/*e*′	11.5 ± 3.2	11.8 ± 3.5	0.66	10.4 ± 2.9	11.6 ± 4.4	0.23
Right heart function						
PAP (mmHg)	31.3 ± 5.7	28.7 ± 9.0	0.33	29.4 ± 6.4	27.8 ± 5.7	0.48
TR Vmax (m/s)	2.26 ± 0.34	2.07 ± 0.57	0.24	2.18 ± 0.36	2.10 ± 0.33	0.69

LV, left ventricular; IVRT, isovolumic relaxation time; E, peak early transmitral flow velocity; *e*′, early diastolic annular velocity; PAP, pulmonary artery pressure; TR Vmax, tricuspid regurgitation velocity maximum.

Table 2 also shows the left ventricular geometric and systolic function, diastolic function and right heart function at the 6-month follow-up visit. Uncontrolled blood pressure during the 6-month follow-up period led to higher LVMI (119 ± 35 vs. 99 ± 25, *P* = 0.02, shown in Figure 3), LAVI (27.6 ± 4.7 vs. 24.8 ± 4.1, *P* = 0.01) and lower *e*′ (6.2 ± 1.6 vs. 7.2 ± 2.2, *P* = 0.04). Whereas the changes in LVMI (−6.2 ± 15.2 vs. 1.8 ± 18.0, *P* = 0.06, shown in Figure 4) or changes in LAVI (−0.4 ± 1.3 vs. −0.3 ± 1.2, *P* = 0.78, data not shown) had no difference between groups during the 6-month follow-up period. LVMI changes were not related to antihypertensive medicine, including combination therapy in 6-month follow up examination (data not shown).

**Figure 2:**
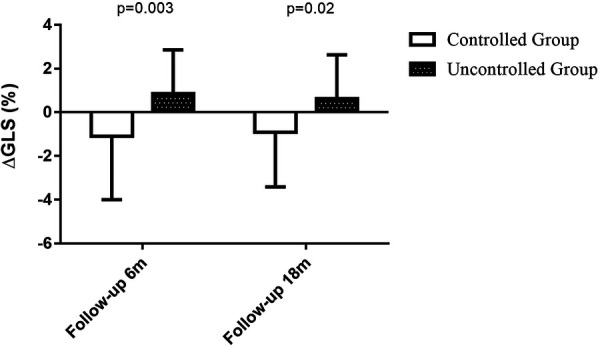
Change in GLS at 6-month follow-up visit and 18-month follow-up visit. During the 6-month follow up period, changes in GLS was −0.9 ± 2.0 in BP controlled group (*n* = 29) vs.1.1 ± 2.9 in uncontrolled group (*n* = 33), *P* = 0.003. During the 18-month follow up period, changes in GLS was −0.6 ± 2.0 in BP controlled group (*n* = 24) vs. 0.9 ± 2.5 in uncontrolled group (*n* = 25), *P* = 0.02.

At the 6-month follow-up visit, GLS was better in BP controlled group than in uncontrolled group (−20.0 ± 2.5 vs. −18.2 ± 2.4, *P* = 0.005) (Figure 1). The changes in GLS of BP controlled group were improved compared to uncontrolled group (−0.9 ± 2.0 vs.1.1 ± 2.9, *P* = 0.003) at the 6-month follow-up examination (Figure 2). Changes in GLS during 6 months were related to BMI (*r* = 0.257, *P* = 0.03), BP control (*r* = 0.370, *P* = 0.002), whereas they were inversely related to age (*r* = −0.299, *P* = 0.01), sex (*r* = −0.248, *P* = 0.03) and systolic BP (*r* = −0.359, *P* = 0.002) in univariate analysis. While in multivariate analysis, changes in GLS only had correlation with BP control (*β *= 0.370, *P* = 0.004). Changes in GLS were unrelated to diabetes mellitus, hyperlipoidemia or diastolic BP during the 6 months follow up period (Table 3).

**Table 3:** Univariate and multivariate linear model of △GLS during 6 months follow-up period.

Variables	Univariate correlation coefficient	*P* value	Multivariate beta-coefficient (SE)	*P* value
Age (years)	−0.299	0.01		
Sex	−0.248	0.03		
Body mass index (kg/m^2^)	0.257	0.03		
Blood pressure control	0.370	0.002	0.370 (0.640)	0.004
Diabetes mellitus	−0.096	0.23		
Hyperlipoidemia	−0.080	0.27		
Systolic blood pressure (mmHg)	−0.359	0.002		
Diastolic blood pressure (mmHg)	−0.130	0.16		

**Figure 3:**
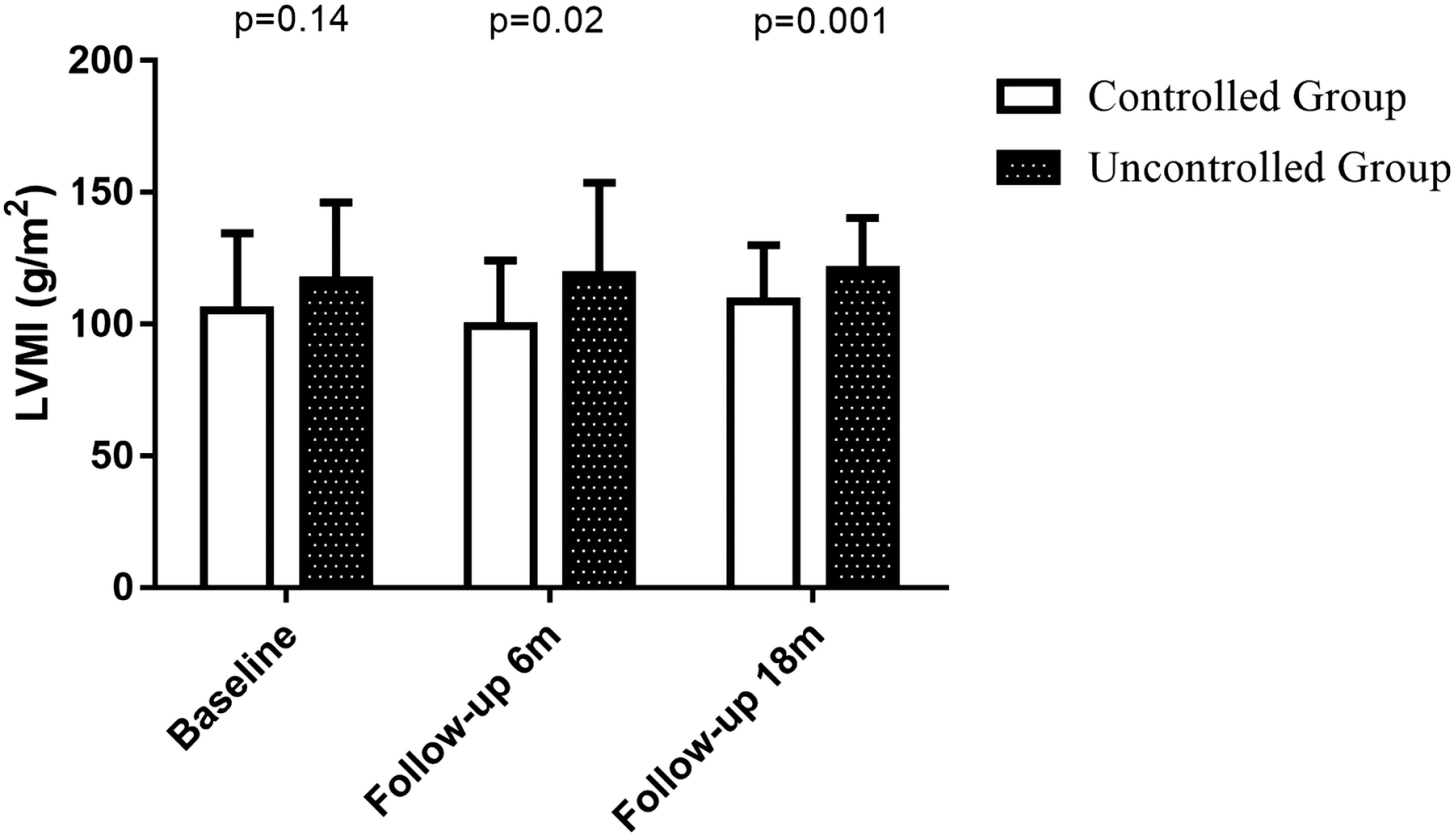
LVMI at baseline, 6-month follow-up period and 18-month follow-up period. LVMI at baseline: controlled group (*n* = 29): 105 ± 29 vs. uncontrolled group (*n* = 33): 117 ± 30, *P* = 0.14; LVMI at 6-month follow-up visit: controlled group (*n* = 29): 99 ± 25 vs. uncontrolled group (*n* = 33): 119 ± 35, *P* = 0.02; LVMI at 18-month follow-up visit: controlled group (*n* = 24): vs. 101.4 ± 21.7 vs. uncontrolled group (*n* = 25): 125.8 ± 27.8, *P* = 0.001.

**Figure 4:**
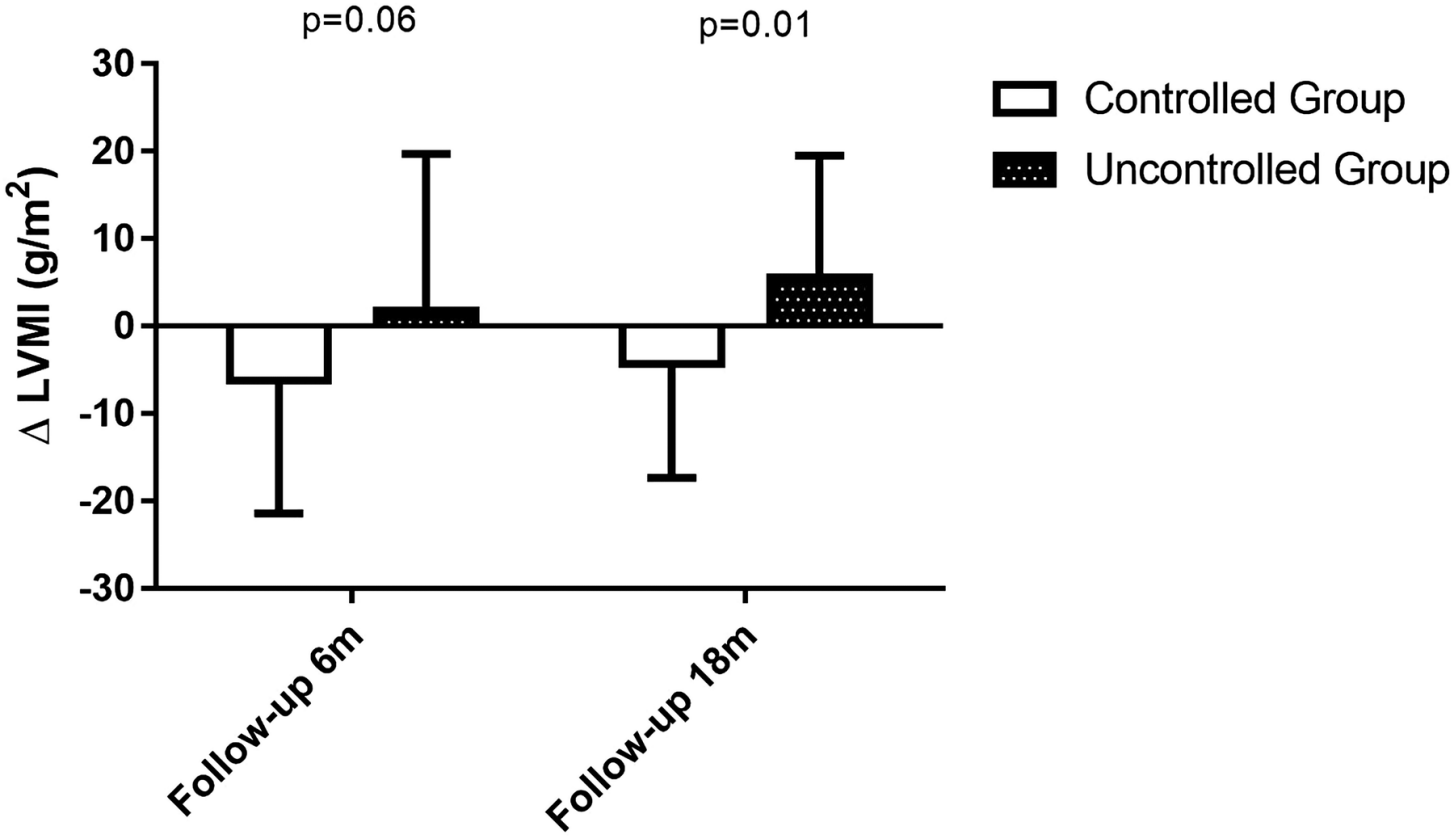
Changes in LVMI at 6-month follow-up period and 18-month follow-up period. During the 6-month follow up period, changes in LVMI was −6.2 ± 15.2 in BP controlled group (*n* = 29) vs. 1.8 ± 18.0 in uncontrolled group (*n* = 33), *P* = 0.06. During the 18-month follow up period, changes in LVMI was −4.3 ± 13.0 in BP controlled group (*n* = 24) vs. 5.6 ± 14.0 in uncontrolled group (*n* = 25), *P* = 0.01.

As shown in Table 4, 49 patients had their 18-month follow-up echocardiographic examinations. According to their BP control during the 12 months follow up period after the second medical visit, the hypertensive patients were classified into controlled group (*n* = 24, 49.0%) and uncontrolled group (*n* = 25, 51.0%). Uncontrolled group had higher interventricular septum (12.0 ± 1.8 vs. 10.6 ± 1.5, *P* = 0.005) and posterior wall thickness (11.2 ± 1.4 vs. 10.1 ± 1.5, *P* = 0.01), higher LVM (223.8 ± 52.5 vs. 193.2 ± 52.8, *P* = 0.048), LVMI (125.8 ± 27.8 vs. 101.4 ± 21.7, *P* = 0.001, shown in Figure 3), relative wall thickness (0.45 ± 0.07 vs. 0.41 ± 0.05, *P* = 0.01) and average *E*/*e*′ (11.6 ± 3.7 vs. 9.7 ± 2.8, *P* = 0.046). There was no difference in heart rate, LAVI, E/A ratio, deceleration time, IVRT, *e*′ or right heart function between groups at the 18-month follow-up visit. Changes in LVMI were significantly higher in BP uncontrolled group than in controlled group at the 18-month follow-up visit (5.6 ± 14.0 vs. −4.3 ± 13.0, *P* = 0.01, shown in Figure 4), but changes in LAVI had no difference between groups (0.3 ± 0.8 vs. 0.4 ± 1.0, *P* = 0.59). In univariate analysis, changes in LVMI were directly related to systolic BP (*r* = 0.426, *P* = 0.001), diastolic BP (*r* = 0.349, *P* = 0.006) and the usage of ACEI or ARB (*r* = 0.280, *P* = 0.025), whereas they were inversely related to BP control (*r* = −0.352, *P* = 0.007) at the 18 months follow-up examination (Table 5). As in multivariate analysis, changes in LVMI only had correlation with systolic BP (*β *= 0.426, *P* = 0.002).

**Table 4:** Patients' echocardiographic characteristics at follow-up 18 m visit.

	Controlled group	Uncontrolled group	*P* value
	(*n* = 24)	(*n* = 25)	
Left ventricular geometric and systolic function parameters			
LV end-diastolic diameter (mm)	49.8 ± 3.5	49.8 ± 4.6	0.99
LV end-systolic diameter (mm)	30.0 ± 3.0	30.0 ± 3.2	0.96
LV end-diastolic volume (ml)	117.7 ± 18.5	118.6 ± 24.2	0.88
LV end-systolic volume (ml)	35.5 ± 8.8	36.0 ± 9.4	0.85
LV ejection fraction (%)	69.9 ± 5.6	69.8 ± 3.8	0.91
Interventricular septum (mm)	10.6 ± 1.5	12.0 ± 1.8	0.005
LV posterior wall (mm)	10.1 ± 1.5	11.2 ± 1.4	0.01
LV mass (g)	193.2 ± 52.8	223.8 ± 52.5	0.048
LV mass index (g/m^2^)	101.4 ± 21.7	125.8 ± 27.8	0.001
Relative wall thickness	0.41 ± 0.05	0.45 ± 0.07	0.01
Heart rate (bpm)	67.8 ± 7.5	67.5 ± 9.9	0.90
Left ventricular diastolic function parameters			
Left atrial volume index (ml/m^−2^)	8.4 ± 1.3	9.2 ± 1.6	0.053
Early: late-wave ratio	0.88 ± 0.19	0.85 ± 0.26	0.60
Early wave deceleration time (ms)	230 ± 45	225 ± 49	0.74
IVRT (ms)	97 ± 15	108 ± 22	0.06
*e*′ (cm/s)	7.3 ± 1.9	6.2 ± 1.9	0.054
*E*/*e*′	9.7 ± 2.8	11.6 ± 3.7	0.046
Right heart function			
PAP (mmHg)	30.8 ± 6.1	28.7 ± 6.2	0.50
TR Vmax (m/s)	2.26 ± 0.34	2.13 ± 0.38	0.17

**Table 5:** Univariate and multivariate linear model of △LVMI during 18 months follow-up period.

Variables	Univariate correlation coefficient	*P* value	Multivariate beta-coefficient (SE)	*P* value
Age (years)	−0.156	0.14		
Sex	0.122	0.20		
Body mass index (kg/m^2^)	−0.070	0.32		
Blood pressure control	−0.352	0.007		
Usage of ACE-I/ARB	0.280	0.025		
Diabetes mellitus	0.162	0.13		
Hyperlipoidemia	0.199	0.08		
Systolic blood pressure (mmHg)	0.426	0.001	0.426 (0.086)	0.002
Diastolic blood pressure (mmHg)	0.349	0.006		

ACE-I, medication with angiotensin-converting enzyme (ACE) inhibitor; ARB, medication with angiotensin receptor blocker; CCB, medication with calcium channel blocker.

At the 18-month follow-up examination, GLS in BP uncontrolled group was worse than that in controlled group (−18.8 ± 3.5 vs. −20.1 ± 3.1, *P* = 0.02) (Figure 1). The changes in GLS of BP controlled group were improved compared to that in uncontrolled group (−0.6 ± 2.0 vs. 0.9 ± 2.5, *P* = 0.02) at the 18-month visit, as shown in Figure 2. Changes in GLS were related to BP control (*r* = 0.324, *P* = 0.01), although it was not related to age, sex, BMI, diabetes mellitus, hyperlipoidemia, or diastolic BP in univariate analysis during the 18 months follow up period. While in multivariate analysis, changes in GLS only had correlation with BP control (*β *= 0.324, *P* = 0.02) (Table 6). Neither was GLS changes related to antihypertensive medicine, including combination therapy in 6- or18- month follow up examination (data not shown).

**Table 6:** Univariate and multivariate linear model of △GLS during 18 months follow-up period.

Variables	Univariate correlation coefficient	*P* value	Multivariate beta-coefficient (SE)	*P* value
Age (years)	−0.054	0.36		
Sex	0.154	0.14		
Body mass index (kg/m^2^)	−0.142	0.16		
Blood pressure control	0.324	0.01	0.324 (0.658)	0.02
Diabetes mellitus	−0.034	0.41		
Hyperlipoidemia	0.048	0.37		
Systolic blood pressure (mmHg)	−0.223	0.06		
Diastolic blood pressure (mmHg)	−0.126	0.19		

## Discussion

This study demonstrated that BP control was associated with echocardiographic progression during 18-month follow-up period in essential hypertensive patients. GLS improvement was significant between BP controlled and uncontrolled patients even in 6-month follow-up period. Our findings especially support the view that GLS was an earlier and subtler marker than conventional LV geometry and function parameters.

Several studies have previously provided independent and incremental prognostic information of GLS for cardiovascular morbidity and mortality (20–22). The improvement of conventional echocardiographic parameters for LV geometry, LV systolic and diastolic function (8–11), as well as LV GLS after antihypertensive treatment (8, 12–16) were widely reported. Moreover, most research focused on the association between antihypertensive medication and GLS progression, instead of BP reduction (23). The consensus about the timing of these changes was rarely reported. To date, the predictive value of BP reduction for LV geometry and function, including GLS progression and the timing of these changes are not well established. This study may be the first to investigate the association between BP reduction and GLS progression, as well as the timing of these changes in uncontrolled primary hypertensive patients.

The role of antihypertensive medications on LV remodeling is always a debated issue. Many studies reported that improvement in GLS was associated with the use of antihypertensive medications, while some studies found that even the antihypertensive classes have different impacts on LV geometry and function (12–16, 24). Cheng et al. revealed that GLS improvement was related to the application of high doses of antihypertensive medications, including valsartan and amlodipine, while it was uncorrelated with blood pressure change in diastolic dysfunction patients with hypertension during 24 weeks follow-up period (12). Motoki et al. found that azelnidipine had beneficial effects on GLS progression comparable to amlodipine in hypertensive patients with LV hypertrophy (13). Vinereanu et al. found that in hypertensive and diabetes patients, indapamide improved GLS compared with hydrochlorothiazide after 6 months of treatment (14). The use of telmisartan improved GLS and LVMI beyond BP reduction in 12 months in hypertension patients (15). Namely, GLS was reported to be more improved in patients treated with valsartan than with amlodipine, with similar BP control and LVMI decrements (24). In addition to the role of BP reduction or antihypertensive medications on LV remodeling, the timing of echocardiographic changes remains uncertain (13). Mizuguchi et al. revealed a significant improvement of GLS observed after 3 months of treatment with telmisartan, and these changes lingered throughout the 9 remaining months of that study (15). Some researchers found that GLS improvement occurred after 6 to 12 months of antihypertensive treatment (13–15). In a 3 year follow up study, Tzortzis et al. showed that both GLS and LVMI were improved with treatment of angiotensin converting enzyme (ACE) inhibitors (ramipril 5 mg) +/− hydrochlorothiazide in newly diagnosed hypertensives (16). Lønnebakken et al. revealed in a 67-month follow-up study that left ventricular hypertrophy regression was related to BP control, independent of number and class of antihypertensive drugs from the Campania Salute Network (25). The present study found that GLS improvement was significant in controlled hypertensive patients compared to uncontrolled ones at the 6-month follow up examination, while LVMI reduction occurred at the 18-month follow up examination. GLS changes or LVMI changes were not related to antihypertensive medicine at neither 6-month nor 18-month follow-up examination after adjustment for other predictive parameters in the present study. The possible reason is that the use of antihypertensive medicine in the present study was not controlled, since we just recorded the patients' blood pressure and medications. Thus, it could be speculated that the impact of blood pressure control was more important to GLS improvement and LVMI reduction than antihypertensive medication class in the treatment of uncontrolled hypertensive patients.

In many studies, GLS improvement was associated with LVMI reduction (10, 11, 13, 26), while other researchers found it independently of LVMI changes (8, 14, 27). The present study showed the independent association between GLS improvement and BP reduction, and GLS improvement was earlier than LVMI reduction. We found that the improvement in GLS was significant between BP controlled and uncontrolled patients in the 6-month follow-up examination, while LVMI reduction was not significant then. The relationship between GLS improvement and LVMI reduction was not significant in either 6-month or 18-month follow-up examination (data not shown). Multivariate analysis also showed that GLS improvement only correlated with BP control during the 6-month and 18-month follow up period. Among parameters of LV mechanics, GLS provides a sensitive and reliable measurement of LV systolic function compared to conventional echocardiographic parameters and its prognostic value has been established in abundant population-based studies (20, 21, 28, 29). The present study confirmed that improvement in GLS was detected before the changes of LVMI and LV ejection fraction. There was no difference in LV ejection fraction between BP controlled and uncontrolled patients during the 6-month and 18-month follow-up period. Cheng et al. found that GLS improvement was associated with BMI and gender (12), but in the present study, we found that it was independent of these factors.

Studies in experimental animals reported that longitudinal strain impairment was significantly related to the level of subendocardial layer fibrosis after adjustment for the corresponding layer wall stress (30). Both collagen I and III were elevated in sub-endocardium in hypertension on the molecular level, which could also result in chamber stiffness (31). It was suggested that the excess of collagen I and III in hypertension could be increasingly synthesized by cardiac fibroblasts and myofibroblasts l (32). Left ventricular fibrosis maybe the result of hemodynamic overload of the left ventricle due to hypertension (32). Therefore, it is easy to understand how blood pressure control can be associated to the improvement of LV geometric and functional properties, including GLS improvement and LVMI reduction. It may suggest that GLS could be a subtle measurement of subendocardial fibrotic changes and may be useful for risk stratification of hypertensive target organ damage, especially in subclinical heart failure (30).

Several important limitations must be acknowledged. First of all, it was a single-center study with a very small population sample from the department of hypertension and outpatient clinic in Ruijin Hospital, Shanghai. We are unable to extrapolate these findings in a multi-centered Chinese population study. Additionally, parasternal short-axis views, which were used to assess circumferential strain, were not included in this study. Moreover, the antihypertensive medicine for the present study was not controlled, and the target of BP control in the present study was 140/90, as many researchers found that LV geometric and functional parameters can be ameliorated in the setting of a lower BP target or targeted antihypertensive treatment (12, 16) More research is needed to evaluate how much different antihypertensive medicines and different BP lowering goals add to GLS and LVMI progression in hypertensive populations.

In conclusion, our study revealed that BP control was associated with the echocardiographic progression in essential hypertensive patients, even after adjustment for some conventional CVD risk factors, including age, sex, diabetes mellitus, hyperlipidemia, BMI, and blood pressure. GLS improvement was significant between BP controlled and uncontrolled patients even in 6-month follow-up period, however, LVMI reduction was not significant until 18-month follow-up examination between groups. Our findings suggest blood pressure control was more important to GLS and LVMI improvement than the usage of antihypertensive medication class in the treatment of hypertension.

## Data Availability

The original contributions presented in the study are included in the article/Supplementary Material, further inquiries can be directed to the corresponding author.
